# Irrigation Rationalization Boosts Wheat (*Triticum aestivum* L.) Yield and Reduces Rust Incidence under Arid Conditions

**DOI:** 10.1155/2021/5535399

**Published:** 2021-09-06

**Authors:** Adnan Alghawry, Attila Yazar, Mustafa Unlu, Yeşim Bozkurt Çolak, Muhammad Aamir Iqbal, Celaleddin Barutcular, Enas M. El-Ballat, Gaber El-Saber Batiha, Abdur Rauf, Fatma M. El-Demerdash, Mohamad Elshafee, Sobhy Sorour, Murat Erman, Mohamed A. El-Esawi, Ayman EL Sabagh

**Affiliations:** ^1^Department of Irrigation Research, Central Highlands Research Station, Dhamar, Yemen; ^2^Department of Irrigation and Agricultural Structures Science, Faculty of Agriculture, Cukurova University, 01330 Adana, Turkey; ^3^Alata Horticultural Research Institute, Water Management Department, Mersin, Turkey; ^4^Department of Agronomy, Faculty of Agriculture, University of Poonch Rawalakot (AJK), Pakistan; ^5^Department of Field Crops, Faculty of Agriculture, University of Çukurova, Turkey; ^6^Botany Department, Faculty of Science, Tanta University, Tanta 31527, Egypt; ^7^Department of Pharmacology and Therapeutics, Faculty of Veterinary Medicine, Damanhour University, Damanhour 22511, Beheira, Egypt; ^8^Department of Chemistry, University of Swabi, Ambar 23430, Pakistan; ^9^Environmental Studies Department, Institute of Graduate Studies and Research, Alexandria University, Egypt; ^10^Department of Agronomy, Faculty of Agriculture, Kafrelsheikh University, Egypt; ^11^Department of Field Crops, Faculty of Agriculture, Siirt University, Turkey

## Abstract

Under changing climate, water scarcity and frequent incidence of diseases like stripe rust pose the biggest threat to sustainable crop production which jeopardizes nutritional security. A study was executed to rationalize crop water requirement and evaluate wheat (*Triticum aestivum* L. cv. Bohoth 3) yield losses by stripe rust infection under irrigated conditions. Seven water treatments included three irrigations in three stages/season (*S*_3_), four irrigations (*S*_4_), and five irrigations (*S*_5_) at the different sensitive growth stages, full (*F*), and two deficit irrigation levels including *D*_1_ = 80% of field capacity (FC) and *D*_2_ = 70% (FC) along with farmers' practice of irrigation as control (*C*). Results revealed that *F* and *D*_1_ boosted grain yield by 31 and 14%. Overall, *F* irrigation regime resulted in the highest grain production (2.93 ton/ha) as well as biomass yield (13.2 ton/ha). However, *D*_2_ had the highest value of grain protein (15.9%) and achieved the highest application efficiency (AE) at midseason (54.6%) and end season (59.6%), and the lowest AE was under *S*_3_. Also, halting irrigation at the milky stage (*S*_5_) led to a significant decrease in irrigation water use efficiency as compared to *D*_1_. However, cutting irrigation at the end of seedling, heading, and milky stages (*S*_3_) caused a significant reduction in *E*_a_, crop water use (ET_a_), and 1000 grain weight in comparison with all other treatments. Regarding yellow rust, *S*_3_ irrigation regime resulted in the lowest incidence of yellow rust infection. The highest irrigation and water use efficiency values were recorded under *D*_1_ (0.79 and 0.59 kg/m^3^), and the lowest values were obtained for control. Hence, the deficit irrigation treatment *D*_1_ could be recommended as the best appropriate strategy to save more water and to improve the water productivity under Yemeni agroclimatic conditions.

## 1. Introduction

Globally, wheat is a major and most important staple food crop that is being grown on a larger area [1], and its demand is projected to be increased by 60% up till 2050 [2–4]. Due to the involvement of subsistence farmers, wheat production also has a major impact on household food and nutritional security. In the Central and West Asia and North Africa (CWANA) region, wheat is the basic staple and food security crop, contributing over 60% of people's daily calorific needs. However, wheat productivity in the region is very low (less than 2.5 t/ha) which is due to the major abiotic (drought, cold, heat, and salinity) and biotic (stripe rust, leaf rust, stem rust, and Hessian fly) stresses [5]. Owing to serious water scarcity and disease incidence, Yemen is heavily dependent on wheat imports.

In many rain-fed environments, shortage of soil moisture often occurs during the most sensitive stages of crop growth (flowering and grain filling) which can severely affect plant growth and yield. The application of limited amounts of water during critical crop growth stages can substantially increase yield and water productivity by reducing the drastic impacts of drought [6, 7]. Also, full irrigation becomes economically unviable for most producers who tend to rely on reducing irrigation level to cover the loss of yield revenue [8, 9]. Supplemental irrigation insensitive growth stages can help allowing the plant to increase its photosynthesis rate and give extra time to translocate the carbohydrate to grains, which could improve grain size and thereby may lead to increase grain yield [10]. Using limited SI during sensitive growth stages can boost WUE and wheat yield [11]. Irrigation level of 80% FC remained equivalent to full irrigation as far as the yield was concerned while lesser deficit irrigation recorded significant yield reduction [12]. The water use efficiency for wheat ranged from 0.44 kg/m^3^ for rain-fed to 1.04 kg/m^3^ for full supplementary irrigation treatment [13]. Contrarily, Farre and Faci [14] reported that water use efficiency decreased with decreasing irrigation levels.

The time and growth stage determination to apply deficit irrigation is critical to obtain comparable yields as that of full irrigation. The quantity of irrigation water is reduced at the beginning and end of crop season because consumptive use is being less [15]. Wheat crop during the maturity stage has low sensitivity to water stress. So, the irrigation process could be stopped [16].

Water application efficiency, which is the ratio of retained moisture in the root zone to the applied irrigation water, can serve as a vital indicator to boost water productivity [17]. In addition, Zhang et al. [18] concluded that deficit irrigation at critical growth stages resulted in higher water application efficiency without a significant loss in grain yield.

Similarly, Tari [19] recommended that water deficit should be applied in milk grain stage of wheat to reduce the yield losses. Water application efficiency might be boosted by applying water at seedling milking stages of wheat [20]. However, another study reported that irrigation at tillering and grain filling stages increased water application efficiency [21]. Full irrigation treatment resulted in high grain and biomass yield of wheat, and this impact differed depending on the irrigation amount and its application stage [22]. Seleiman et al. [23] stated that less irrigation and water stress increased protein content by 11.20-13.40% as compared to full irrigation. It was reported that irrigation applied in the milk stage increased the protein ratios, where the highest protein ratio was obtained under the moisture conditions of the full irrigation at milk stage of wheat [19]. Likewise, Noorka and Silva [24] found that, under normal irrigation conditions, the protein contents of wheat ranged from 11.2 to 13.78%, while under water stress condition, it ranged from 12.47 to 13.92%. There was a positive relationship between grain yield and morphological traits (spike length, peduncle length, awn length, and grains/spike) under water stress conditions [25]. Moisture stress could reduce biomass, grains per spike, and grain size at any stage when it occurs. Thus, the overall effect of moisture stress depends on the intensity and length of stress [26].

Yellow rust caused by *Puccinia striiformis* f. sp. *tritici* (Pst) and leaf rust caused by *P. recondite* f. sp. *tritici* (Prt) are two kinds of economically important airborne diseases that could infect wheat worldwide [27, 28]. However, wheat stripe rust (also called yellow rust) is the most destructive disease worldwide and might result in a complete crop loss [29, 30]. The rust disease might spread to Yemen and Sudan in 2006 and further spread towards North Africa and the Middle East [31]. Yield loss due to yellow rust natural infection at highland areas ranged from 27% to 35% [32]. Similarly, Shaalan et al. [33] found that the ability of Bohoth 3 variety for infection in yellow rust was 20MS (moderately susceptible), stem rust 30S (susceptible), and leaf rust (10MS). The infection of wheat by yellow rust appears in winter season, particularly at the temperature of 5-22°C with an increasing humidity [34]. Tolerance to yellow rust is one of the most important targets of wheat breeding programs in all wheat-growing regions of the world [35]. Nowadays, wheat yellow rust is the major obstacle to a stable and high yield of wheat [36].

The experiment is aimed at rationalizing water requirement of the wheat crop through calculation of the optimal water requirement, as well as rationalizing water through using deficit irrigation techniques. It is also aimed at studying the irrigation effect at the critical stages on the grain production. Moreover, it evaluated the effect of full, deficit irrigation and irrigation in sensitive growth stages on water characteristics and crop characteristics. Lastly, the incidence of wheat yellow rust infection to different irrigation schedule systems was also quantified.

## 2. Materials and Methods

### 2.1. Experimental Site and Procedures

The experiment was carried out at the research farm of Central Highland Station (14°54′N latitudes, 44°41′E longitudes, and altitude of 2421 m) during the winter season of 2011 using wheat crop variety Bohoth 3. The climatic measurements were taken: maximum and minimum air temperature, relative humidity, wind speed, sunshine hours, rainfall, and estimated reference evapotranspiration (ET_o_) mm/month along with long-term means are presented in Table 1.

To determine soil physicochemical characteristics, soil samples were taken from three depths (0-30, 30-60, and 60-90 cm) and were subsequently analyzed for pH and electrical conductivity (EC), acidity, and salinity of irrigation water (Table 2).

The experiment was performed in the field in January 2011. The nitrogen was applied as a urea fertilizer (120 kg ha^−1^) in two equal splits at planting and tillering stages, while phosphorous was added as triple superphosphate fertilizer (100 kg ha^−1^) by following Yemeni research recommendations [37]. The yellow rust infection appeared at the grain filling stage where it was evaluated in two different periods, and the harvesting was in May 2011 (4 m^2^/experimental unit).

The disease severity of stripe (yellow) rust was measured according to the modified Cobb scale [38]; however, the infection type of stripe rust was recorded according to Akhtar et al. [39] as shown in Table 3.

### 2.2. Experimental Design and Treatments

The experiment included seven water treatments: the first was full irrigation level 100%, two treatments under deficit irrigation *D*_1_ and *D*_2_ (80, 70% FC), three treatments *S*_3_, *S*_4_, and *S*_5_ applying irrigations at different growth stages of the crop (planting, full seedling, tillering, elongation, heading, and milky stage), *S*_5_ applied five irrigations at the above-mentioned stages except for the milky stage, and four irrigation (*S*_4_) except one irrigation of *S*_5_ at the seedling stage, three irrigations (*S*_3_) at planting, tillering and stem elongation stages, and the seventh treatment under farmers' conditions.  Table 4 shows growth and development stages for a wheat variety Bohoth 3. Plot dimensions were 4.2 m in length and 3.5 m in width, with an area of 14.7m^2^.

### 2.3. Meteorological Data

The average annual temperature in Dhamar region remained at 16.1°C, while rainfall was 377 mm. In the initial and development stage of the winter wheat season, rainfall was scarce (9-33 mm) during the months of January, February, and March. However, at the advanced growth stages, the monthly precipitation was increased during April and May. The mean monthly temperature during the wheat season varied between 12.25 and 17.8°C. The long-term historical climatic data have been presented in Table 1.

### 2.4. Irrigation Scheduling

Irrigations were scheduled based on the climatic data, the soil moisture, and the sensitive growth stages using a computer software program (Crop-Wat). Net irrigation requirement was adjusted for application efficiency of furrow method (60%) by dividing the resulted irrigation quantity by the irrigation efficiency as shown in Table 5. The irrigation quantity of experimental units was measured using a water meter. The soil moisture content was monitored prior to each irrigation using the gravimetric method at the different stages in the active root depth.

Irrigation was applied to restore the moisture deficit in the root zone (90 cm depth) when 50% of available water had been depleted. The full water requirement was 402 mm per season, and deficit irrigations included *D*_1_ = 322 mm per season and *D*_2_ = 282 mm per season. In addition, irrigations at the growth stages of wheat were *S*_5_ = 346, *S*_4_ = 314, and *S*_3_ = 213 mm/season (Table 5). The total rainfall during the growing season of wheat amounted to 72.7 mm. It was noticed that applied water at the start and end season was lower [15], as the irrigation at maturity stage was stopped [16]. The irrigation quantity applied by the farmer was 566 mm/season in the winter season.

Water use efficiency (WUE) and irrigation water use efficiency (IWUE) in kg/m^3^ were calculated as previously reported [40]:

(1) $$\begin{array}{c} \text{WUE}=\frac{\text{GY}}{\text{ET}*10},\\ \text{IWUE}=\frac{\text{GY}}{I*10}, \end{array}$$

where GY is the grain yield (kg/ha) and *I* is the added water amount during the season (mm).

Water application efficiency was calculated according to the following relation [41]:

(2) $$\begin{array}{c} E_{\text{a}}=\frac{R_{\text{z}}}{\forall }\times 100, \end{array}$$

where *E*_a_ is the water application efficiency (%), *R*_Z_ is the total water stored in root zone depth (mm), ∀ is the added water amount to each treatment (mm), and *R*_z_ is the value calculated according to the last reference.

(3) $$\begin{array}{c} R_{\text{Z}}=\frac{\Delta \theta_{W}\times \rho_{b}\times D}{100}, \end{array}$$

where Δ*θ*_*W*_ is the difference of the moisture content before and after irrigation (%), *D* is the active root depth (0-90 cm), and *ρ*_*b*_ is the soil bulk density (g/cm^3^).

### 2.5. Statistical Analysis

The results were statistically analyzed using SAS (Statistical Analysis System, Version 9.00 TS level 00M0 XP_PRO platform), and a comparison between treatment means was set up through Duncan's Multiple Range Test (DMRT) at a 5% probability level.

## 3. Results and Discussions

### 3.1. Water Characteristics

#### 3.1.1. Evapotranspiration (ET_a_) and Water Saving (%)

The full and deficit irrigation treatments *F*, *D*_1_, and *D*_2_ saved water by 28, 43, and 50% in comparison with the control. The treatments in sensitive growth stages of *S*_3_, *S*_4_, and *S*_5_ achieved water savings of 62, 44, and 38% compared to the amount of water, which was added by the farmers.

In general, ET_a_ increased with increasing irrigation quantity and the seasonal crop ET_a_ was higher at higher irrigation levels than the deficit irrigation levels. Seasonal ET_a_ in 2011 ranged from 689 mm under the farmer's practice to 314 mm for *S*_3_ treatment (Table 6). The seasonal crop evapotranspiration of full irrigation treatment was higher than irrigation in sensitive growth stages of *S*_5_, *S*_4_, and *S*_3_ by 13.7, 20.7, and 63.3% in the growing season.

The five-irrigation treatment (*S*_5_) dominated significantly on other treatments (except full irrigation and farmer practice) in ET_a_ with the rate of 451 mm/season. The grain yield differed depending on the irrigation scheduling and wheat growth stage; these results are in agreement with previous findings [18].

#### 3.1.2. Irrigation and Water Use Efficiency (IWUE–WUE)

Regarding water use efficiency, significant differences were found among irrigation regimes owing to a considerable decrease in grain production. The deficit irrigation (*D*_1_ = 80% FC) outperformed other irrigation regimes by achieving water use efficiency values of 0.79 and 0.59 kg/m^3^, respectively, followed by *F* and *D*_2_ treatments. The highest grain production by *D*_1_ might be attributed to its superior performance compared to other irrigation levels and control. The analysis of results in the study year revealed that the treatment received irrigation at 5 growth stages. *S*_5_ increased IWUE and WUE by (8.6, 14.5%) and (11.6, 29.7%) as compared by *S*_4_ and *S*_3_ treatments, respectively. IWUE variations of the treatments were higher than WUE, depending on the relative relationships between grain yield and applied water amount (Table 6). Those findings are in contradiction with the conclusions of Farre and Faci [14], who stated that water use efficiency decreased with decreasing irrigation. Similar studies reported that the highest WUE occurred in DI_75_ instead of full irrigation because the relatively lower yield under deficit irrigation and IWUE increased with decreasing irrigation amount and/or water use [11, 13, 42–44]. On the other hand, the results are not in line with the findings of Ul-Allah et al. [45], who stated that WUE under water deficit conditions was higher in comparison to well-irrigated conditions.

#### 3.1.3. Water Application Efficiency in the Midseason and End Season Stages (Ea_mid_-Ea_end_)

Water application efficiency in the midseason was significantly differenced among irrigation levels (Table 6), where 70% of FC remained unmatched (54.6%) at sensitive stages. Applying smaller water amounts can reduce deep percolation losses and improve this irrigation efficiency. Hence, the increase of application efficiency under deficit irrigation treatment 70% (*D*_2_) was due to increased water stress that prevented water loss to deeper layers of soil compared to other treatments. These results are consistent with previous findings [46].

Through the end season stage, it was observed that the differences in the moisture content before and after irrigation became lower compared to the midseason stage (Table 6). The treatments of deficit and full irrigation and *S*_5_ dominated significantly over the control. The deficit irrigation, especially *D*_2_ achieved the highest application efficiency (59.6%) at the end season, while the corresponding value for control treatment was 41.5%.

### 3.2. Crop Characteristics

#### 3.2.1. Yield and Yield Components

Full irrigation achieved the highest grain yield (2.93 ton/ha) and the lowest value under irrigation in three growth stages *S*_3_ (1.18 ton/ha) during the growing season. Similar results were reported by Sezen and Yazar [47], who reported the highest grain yield of wheat was attained from full irrigation and the lowest yield was obtained from the rain-fed treatment. These results are in line with the findings of previous studies [22, 44, 48] which reported that grain yield reflected strongly high sensitivity to water stress conditions, where full conventional irrigation treatment achieved significantly greater grain yield compared to full irrigation at flowering and grain filling stages.

Data regarding deficit irrigation at different growth stages showed that water deficiency at planting, tillering, and stem elongation stages (*S*_3_) reduced wheat yield by 35.5 and 46.1%, respectively. The full and deficit irrigation 80% (*D*_1_) surpassed other irrigation regimes in terms of grain yield. The reduction of grain yield under the irrigation treatment *S*_3_ could be due to the crop sensitivity to water stress during flowering and grain filling periods in which this treatment of *S*_3_ was not irrigated in these periods. The results are in agreement with the findings of Tari [19], who recommended that water deficit should be applied in the grain filling stage of wheat to reduce the yield losses.

Moreover, it was noted that irrigation at the grain filling stage (*S*_5_) enhanced total biomass yield by 10.4 and 23.4% as compared to *S*_4_ and *S*_3_, respectively. It was noticed that decreasing irrigation requirement from 322 mm in *D*_1_ to 282 mm in *D*_2_ led to the remarkable difference in biomass yield that equaled to 1.4 ton/ha. The full irrigation treatment produced the highest value of 13.2 ton/ha, whereas the dry matter yield value reached to 11.9 ton/ha under control. The reason for the enhancement of the biomass yield under the full irrigation might be due to the capability of this treatment to keep the soil moisture for a long period which makes the roots spread in a larger area and deeper depth in soil. These results were in agreement with the previously reported findings [12].

Weight of 1000 grain is one of the most prime factors, which contribute to grain yield under irrigated and rain-fed conditions. The full irrigation (*F*) significantly enhanced 1000 grain weight (9.3%) in comparison to farmer's practice; however, the control treatment had higher 1000 grain weight than the *S*_5_ treatment with a slight difference of 1.8%. No significant difference was observed between deficit irrigation treatments, where the treatment *D*_1_ recorded 45.5 g. However, significant differences were recorded between irrigation treatments at sensitive growth stages.

The findings of the present study revealed that there is a strong positive relationship between 1000 grain weight and grain yield of wheat. In *S*_4_ and *D*_2_ treatments, grain yield reduction reached to 28.5 and 17.9% compared to deficit irrigation *D*_1_ which resulted in reductions by 22.4 and 6.3%, respectively. The response of 1000 grain weight differed depending on water application to wheat. Similar results were confirmed by previous studies [20, 49, 50] which stated that the response of 1000 grain weight to irrigation strategies differed depending on the irrigation levels and at which wheat growth stage it was applied.

The results of several grains per spike are presented in Table 7. The decrease in grain yield was a result of the decrease in yield components. The results of the study indicated that a decrease of water deficit from full irrigation towards 70% led to an obvious reduction in grain yield and grain number/spike by 28.3 and 10.3%. There was no significant difference between the deficit irrigation treatments and irrigation regimes in sensitive growth stages where the values of this character were with a medium average under the treatments of *D*_1_, *C*, and *S*_5_. The full irrigation treatment appeared with a slight increase of 44.55 grain/spike. These results are in a general agreement with the previous findings which reported that grain yield was directly correlated to yield attributes [25, 26].

#### 3.2.2. Protein Ratio and Yellow Rust Infection

Water stress resulted in higher protein content as 70% FC recorded the highest protein (15.9%) which was significantly higher than control (13.1%). On the other hand, water deficit under 70% treatment *D*_2_ reduced the grain yield to 28.3% of full irrigated treatment, but it increased protein content by 16.9% over the full irrigation. Application of irrigation in grain filling stage under *D*_2_ and *D*_1_ caused an increase in protein content about 13.5 and 2.8% over the nonirrigation at this stage, *S*_5_. These results are in contrast to the findings of Tari [19], who found that irrigation applied at milking stage increased the protein ratios, while the maximum protein content was recorded for full irrigation. It can be concluded that there is a negative relationship between grain protein content and yield as well as irrigation quantity and protein content. The results also are in agreement with the previous conclusions [23, 24, 49] that reported a positive response between the quality parameters such as protein content, wet gluten content and sedimentation volume, and water stress.

The incidence of yellow rust started to appear during the grain filling stage, and its severity multiplied with increasing water application (Table 7). The type of disease reaction (infection type) was described as MS (moderately susceptible). The farmer irrigation practice (which recorded the highest irrigation amount) dominated significantly on all irrigation treatments (except *F* and *D*_1_ treatments) with an average severity of 58.3%; however, the lowest rust severity of 13.3% was recorded in *S*_3_ irrigation treatment.

It was observed that the low irrigation frequency treatment of stopping irrigation during heading and seedling stages (*S*_3_) decreased the yellow rust infection with differences of 25.3 and 40% compared to irrigation treatments of *S*_5_ and *F*. The increased irrigation amounts under full irrigation treatment (*F*) or high irrigation frequency (*S*_5_) resulted in higher soil content in the active root zone area and lead to a noticeable increase in humidity around the plants, consequently raising the rust infection ratio. Similar results were reported by Gao et al. [51]. It means that increasing the applied water quantity led to an increase in grain yield and generated good conditions to increase the stripe rust infection. These results are in conformation with previous reports [32, 34, 36] whereby irrigation rationalization and use of high resistant genotypes to yellow rust were recommended. On the other hand, this topic still needs more future studies for better understanding the relationship between irrigation and rust infection and other factors such as physiological and environmental factors and their effects on wheat production.

### 3.3. The Relationships between Yield, Yield Components, Applied Water, Protein Content, and Stripe Rust Infection

**Figure 1:**
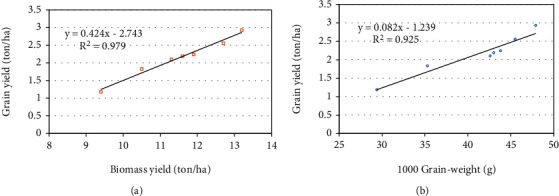
The relationships between (a) grain yield–biomass yield and (b) grain yield–1000 grain weight.

The relationships between grain yield and yield components (biomass yield and 1000 grain weight) are depicted in Figures 1(a) and 1(b)). In general, the biomass and thousand grain weight correlated significantly and positively (*P* < 0.01) with grain yield. The linear relation reflects the strong effect of yield components on developing wheat grain yield. The simple regression equation of grain yield showed a high determination coefficient of 0.97 and 0.92 against biomass yield and 1000 grain weight. Similar results were reported by Ahmadizadeh et al. [52] who stated that wheat grain yield showed positive and significant correlation with 1000 grain weight under normal and stress conditions. These results also are in agreement with other studies [53, 54], which stated that increasing grain yield was noted with increasing biomass yield.

**Figure 2:**
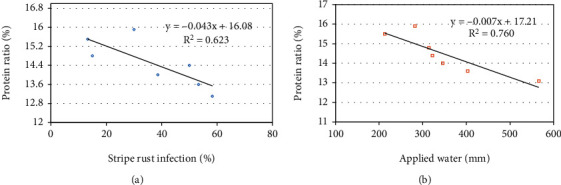
The relations between (a) stripe rust infection–protein content and (b) protein content–applied water.

Regarding the regression analysis, stripe rust infection was considered as an independent variable. So, protein content was graphed as a function of stripe rust infection (Figure 2(a)). For the response of protein content to stripe rust severity, a linear and negative correlation was observed (*r* = −0.78) which means that when the disease severity of stripe rust increased, the protein content decreased. The results can be used to produce high grain protein quality via using resistant wheat varieties to stripe rust disease.

The statistical analysis indicated that negative and significant correlations were detected and the grain quality of protein content responded well to applied water quantity in the root zone with a correlation coefficient value of -0.87 (Figure 2(b)). Similar findings were stated by Oury and Godlin [55], who reported that the protein contents have significant negative correlations with grain yield and thousand kernel weights under normal and stress conditions. It is known that yield reduction that generally occurs under water stress conditions is generally associated with an increase in the protein content [56–58].

## 4. Conclusions

The study was conducted to rationalize irrigation water and reduce the incidence of yellow rust under arid conditions. It was inferred that full irrigation achieved water saving of 28% and significantly increased grain yield (31%) compared to control. Among deficit irrigation regimes, 70% FC achieved water saving of 43% with a 14% increase in grain yield. The deficit irrigation treatments *D*_1_ and *D*_2_ attained water saving by 20 and 30%, respectively, in comparison with full irrigation; however, the irrigation treatments at critical stages of *S*_3_, *S*_4_, and *S*_5_ saved water by 47, 22, and 14%, respectively, in comparison with the full irrigation. Deficit irrigation *D*_1_ resulted in the highest irrigation and water use efficiency. Stopping irrigation at the milky stage (*S*_5_) led to a significant reduction in application efficiency at midseason and end season in comparison with the deficit and full irrigation. However, irrigation regimes at sensitive stages of *S*_3_ and *S*_4_ imparted resistance to rust which recorded the lowest severity infection by yellow rust in comparison with the other treatments.

## Figures and Tables

**Table 1:** Climatic data during the wheat experiment in 2011 and long-term (1985-2015).

	Growing season 2011				
Climatic elements/months	January	February	March	April	May
Max. temperature (°C)	21.5	22.8	24.4	25.6	25
Min. temperature (°C)	3	5.1	8.1	9.9	10.7
Average temperature (°C)	12.2	13.9	16.2	17.7	17.8
Relative humidity (%)	57	56	54	54	61
Wind speed (km/day)	110.3	146.5	125	168.4	166.4
Sunshine (hour/day)	10.3	10.2	7.1	9.5	8.8
Rainfall (mm/month)	9	0	0	52.6	64.6
Reference evapotranspiration (ET_o_) (mm/month)	82	102	106	133	124
Climatic elements/months	Long-term average (1985-2015)				
Max. temperature (°C)	21.2	22.8	24.9	25.1	28
Min. temperature (°C)	2	3.4	6.3	8.7	9.9
Average temperature (°C)	11.6	13.1	15.6	16.9	18.95
Rainfall (mm/month)	7	8	18	50	45

**Table 2:** Soil characteristics in the research farm.

Depth (cm)	Texture class	pH	EC dS/m	CaCO_3_ %	*ρ*_*b*_^∗^ (g/cm^3^)
0-30	L	7.6	0.71	2.76	1.30
30-60	CL	7.8	0.49	2.67	1.32
60-90	L	7.8	0.33	15.3	1.37
Mean		7.73	0.51	6.91	1.33

^∗^Bulk density.

**Table 3:** The resistant type of stripe rust based on the modified scale.

Type of resistant (reaction)	Response value
No. disease (O)	0.0
Resistant (R)	0.2
Resistant to moderately resistant (RMR)	0.3
Moderately resistant (MR)	0.4
Moderately resistant to moderately susceptible (MRMS)	0.6
Moderately susceptible (MS)	0.8
Moderately susceptible to susceptible (MSS)	0.9
Susceptible (S)	1.0

**Table 4:** The crop development stages of wheat (Bohoth 3).

Months	January		February	March		April-May	
Irrigation No.	1	2	3	4	5	6	
Days after sowing	0	(23–77)	(42–64)	(58–63)	(78–81)	(96-99)	(123–130)
Growth stages	Planting	Seedling	Tillering	Elongation	Heading	Milking	Harvesting
Development stage	Initial		Crop development	Midseason		End season	

**Table 5:** Irrigation quantity values for different water treatments.

Irrigation no.	1	2	3	4	5	6	Water requirement (mm/season)
Deficit irrigation							
(1) (*D*_1_) 80%	14	26	66	90	81	45	322
(2) (*D*_2_) 70%	12	23	57	79	71	40	282
(3) (*F*) 100%	18	32	82	113	101	57	403
Irrigation at sensitive stages							
(4) 3 irrigations	18	—	82	113	—	—	213
(5) 4 irrigations	18	—	82	113	101	—	314
(6) 5 irrigations	18	32	82	113	101	—	346
(7) Control (winter season)	94.4	94.4	94.4	94.4	94.4	94.4	566

**Table 6:** The relationship between the irrigation treatments and water characteristics of wheat.

Water treatments/water characteristics	Irrigation mm	ET_a_ mm *I* + *P* ± ∆*S*^∗^	IWUE (kg/m^3^)	WUE (kg/m^3^)	Ea_mid_ (%)	Ea_end_ (%)	Saving water (%)
(1) *D*_1_ (80%)	322	433^d^	0.79^a^	0.59^a^	51.2^b^	57.1^b^	43
(2) *D*_2_ (70%)	282	389^f^	0.74^ab^	0.53^a^	54.6^a^	59.6^a^	50
(3) *F* (100%)	403	513^b^	0.72^ab^	0.57^a^	43.2^c^	46.9^c^	28
(4) 3 irrigations (*S*_3_)	213	314^g^	0.55^bc^	0.37^ab^	31.8^f^	32.7^g^	62
(5) 4 irrigations (*S*_4_)	314	425^e^	0.58^bc^	0.43^ab^	33.8^e^	36.1^f^	44
(6) 5 irrigations (*S*_5_)	346	451^c^	0.63^b^	0.48^ab^	33.7^e^	45.0^d^	38
(7) Control (*C*)	566	689^a^	0.39^c^	0.32^b^	37.8^d^	41.5^e^	—

The similar letters mean that treatments do not have significant differences; ^∗^ ± ∆*S* is change in soil water storage.

**Table 7:** Yield and yield components, protein ratio, and yellow rust infection in different irrigation treatments.

Yield characteristics/treatments	Grain yield (ton/ha)	Biomass yield (ton/ha)	1000 grain weight (g)	No. of grains/spike	Relative yield (%)	Protein ratio (%)	Yellow rust infection (%)
(1) *D*_1_ (80%)	2.56^a^^∗^	12.7^ab^	45.5^ab^	44.4	14	14.4^d^	50^ab^
(2) *D*_2_ (70%)	2.1^ab^	11.3^ab^	42.6^b^	39.9	-6	15.9^a^	30^bc^
(3) F (100%)	2.93^a^	13.2^a^	47.9^a^	44.5	31	13.6^f^	53.3^ab^
(4) 3 irrigations (*S*_3_)	1.18^b^	9.4^b^	29.4^d^	38.2	-47	15.5^b^	13.3^c^
(5) 4 irrigations (*S*_4_)	1.83^ab^	10.5^ab^	35.3^c^	39.1	-18	14.8^c^	15^c^
(6) 5 irrigations (*S*_5_)	2.19^ab^	11.6^ab^	43.0^b^	42.9	-2	14^e^	38.6^b^
(7) Control (*C*)	2.24^ab^	11.9^ab^	43.8^b^	44.4	—	13.1^g^	58.3^a^

^∗^The similar letters-treatments have no significant differences.

## Data Availability

All the data supporting this study are included in the article.

## References

[1] Chamekh Z., Karmous C., Ayadi S. (2017). Comparative performance of *δ*13C, ion accumulation and agronomic parameters for phenotyping durum wheat genotypes under various irrigation water salinities. *Annals Applied Biology*.

[2] Siddiqui M. H., Iqbal M. A., Naeem W., Hussain I., Khaliq A. (2019). Bio-economic viability of rainfed wheat (*Triticum aestivum L*.) cultivars under integrated fertilization regimes in Pakistan. *Custos e Agronegocio*.

[3] Iqbal M. A., Hussain I., Siddiqui M. H., Ali E., Ahmad Z. (2018). Probing profitability of irrigated and rainfed bread wheat (*Triticum aestivum L*.) crops under foliage applied sorghum and moringa extracts in Pakistan. *Custos e Agronegocio*.

[4] Savadi S., Prasad P., Kashyap P. L., Bhardwaj S. C. (2018). Molecular breeding technologies and strategies for rust resistance in wheat (*Triticum aestivum*) for sustained food security. *Plant Pathology Journal*.

[5] International Centre for Agricultural Research in Dry Areas (ICARDA), Rabat Morocco, Tadesse W., Halila H. (2017). Role of sustainable wheat production to ensure food security in the CWANA region. *Journal of Experimental Biology & Agricultural Sciences*.

[6] Oweis T., Hachum A. (2012). *Supplemental Irrigation, a Highly Efficient Water-Use Practice*.

[7] Alexander R. (2005). *Crop production under deficit irrigation, [M.S. thesis]*.

[8] Payero J. O., Tarkalson D. D., Irmak S., Davison D., Petersen J. L. (2008). Effect of irrigation amounts applied with subsurface drip irrigation on corn evapotranspiration, yield, water use efficiency, and dry matter production in a semiarid climate. *Agricultural Water Management*.

[9] Payero J. O., Tarkalson D. D., Irmak S., Davison D., Petersen J. L. (2009). Effect of timing of a deficit-irrigation allocation on corn evapotranspiration, yield, water use efficiency and dry mass. *Agricultural Water Management*.

[10] Ayed S., Rezgui M., Othmani A. (2017). Response of Tunisian durum (Triticum turgidum ssp. durum) and bread (*Triticum aestivum L*.) wheats to water stress. *Agrociencia*.

[11] Wang B., Liu D. L., Asseng S., Macadam I., Yu Q. (2017). Modelling wheat yield change under CO_2_ increase, heat and water stress in relation to plant available water capacity in eastern Australia. *European Journal of Agronomy*.

[12] Padhi J., Payero J. O., Misra R. K. (2010). Measuring the effect of water stress on wheat evapotranpiration. *Australian Irrigation Conference and Exibition 2010: One Water Many Futures*.

[13] Somme G., Oweis T., El Omar F., Hachum A., Shayeb R., Jooni N. (2005). *Rain-fed wheat productivity with supplemental irrigation in Al Hasakeh, northern Syria*.

[14] Farré I., Faci J. I. (2006). Comparative response of maize (Zea mays L.) and sorghum (Sorghum bicolor L. Moench) to deficit irrigation in a Mediterranean environment. *Agricultural Water Management*.

[15] Raes D., Sahli A., van Looij J., Ben Mechlia N., Persoons E. (2000). Charts for guiding irrigation in real time. *Irrigation and Drainage Systems*.

[16] Kang S., Zhang L., Liang Y., Hu X., Cai H., Gu X. (2002). Effects of limited irrigation on yield and water use efficiency of winter wheat in the loess plateau of China. *Agricultural Water Management*.

[17] Hsiao T. C., Steduto A. E., Fereres P., Elias A. E. (2007). A systematic and quantitative approach to improve water use efficiency in agriculture. *Irrigation Science*.

[18] Zhang X., Li Z., Wang Y. (2000). *Management of supplemental irrigation of winter wheat for maximum profit. Natural resources Management and Environment Department*.

[19] Tari A. F. (2016). The effects of different deficit irrigation strategies on yield, quality, and water-use efficiencies of wheat under semi-arid conditions. *Agricultural Water Management*.

[20] Kanshaw E., Al-shawa F. (2007). The effect of supplemental irrigation on productivity of durum wheat (*Triticum Durum* L.) in the Kenitra province. *Damascus University Journal of Agricultural Science*.

[21] Husman S. H., Ottman M. J., Wegener R. J., Rogers M. T. (2000). *Durum Response to Soil Water Depletion Levels*.

[22] Li J., Wang Y., Zhang M. (2019). Optimized micro-sprinkling irrigation scheduling improves grain yield by increasing the uptake and utilization of water and nitrogen during grain filling in winter wheat. *Agricultural Water Management*.

[23] Seleiman M., Abdel-aal S., Ibrahim M., Zahran G. (2011). Productivity, grain and dough quality of bread wheat grown with different water regimes. *Journal of Agronomy and Crop Science*.

[24] Noorka I. R., Teixeira da Silva J. A. (2012). Mechanistic insight of water stress induced aggregation in wheat (Triticum aestivum L.) quality: the protein paradigm shift. *Notulae Scientia Biologicae*.

[25] Villegas D., Aparico N., Royo C. (2001). Biomass accumulation and main stem elongation of durum wheat grown under Mediterranean conditions. *Annals of Botany*.

[26] Bukhat N. M. (2005). *Studies in yield and yield associated traits of wheat (Triticum aestivum L.) genotypes under drought conditions, [M. S. Thesis]*.

[27] Wellings C. R. (2011). Global status of stripe rust: a review of historical and current threats. *Euphytica*.

[28] Huerta-Espino J., Singh R. P., Germán S. (2011). Global status of wheat leaf rust caused by Puccinia triticina. *Euphytica*.

[29] Liu X., Huang C., Sun Z., Liang J., Luo Y., Ma Z. (2011). Analysis of population structure of Puccinia striiformis in Yunnan Province of China by using AFLP. *European Journal of Plant Pathology*.

[30] Huang C., Sun Z., Wang H., Luo Y., Ma Z. (2012). Effects of wheat cultivar mixtures on stripe rust: a meta-analysis on field trials. *Crop Protection*.

[31] Singh R., Hodson D., Huerta-Espino J. (2008). Will Stem Rust Destroy the World's Wheat Crop?. *Advances in Agronomy*.

[32] Eisa M., Alsaadi M., Albasha R. (2014). *Wheat stripe rust status in Yemen: an overview. In: Abstracts, Second International Wheat Stripe Rust Symposium*.

[33] Shaalan S. A., Malik A. A., AlHusseini M., Aljunaid A. (2010). *Evaluation the Productivity Performance of Improved Variety (Sonileka) Compared to Released Varieties*.

[34] Roelfs A. P., Singh R. P., Sarri E. E. (1995). *Rusts Diseases of Wheat: Concepts and Methods of Diseases Management*.

[35] Akfirat S. F., Aydin Y., Ertugrul F. (2010). A microsatellite marker for yellow rust resistance in wheat. *Cereal Research Communications*.

[36] Chen W., Wellings C., Chen X., Kang Z., Liu T. (2014). Wheat stripe (yellow) rust caused by Puccinia striiformis f. sp. Tritici. *Molecular Plant Pathology*.

[37] Mukred A. O. (1998). *Agricultural Directory of Central Heighlands - Agricultural Sector Management Support - Extension, Training Component*.

[38] Peterson R. F., Campbell A. B., Hannah A. E. (1948). A diagrammatic scale for estimating rust severity on leaves and stems of cereals. *Canadian Journal of Research Section C Botanical Sciences*.

[39] Akhtar M. A., Ahmad I., Mirza J. I., Rattu A. R., Hakro A. A., Jaffery A. H. (2002). Evaluation of candidate lines against stripe and leaf rusts under national uniform wheat and barley yield trial 2000-2001. *Asian Journal of Plant Science*.

[40] Zhang H., Yu C., Kong X. (2018). Progressive integrative crop managements increase grain yield, nitrogen use efficiency and irrigation water productivity in rice. *Field Crops Research*.

[41] James L. G. (1988). *Principles of Farm Irrigation System Design*.

[42] Yazar A., Gökçel F., Sezen M. S. (2009). Corn yield response to partial rootzone drying and deficit irrigation strategies applied with drip system. *Plant, Soil and Environment*.

[43] Zhang D., Li R., Batchelor W. D., Ju H., Li Y. (2018). Evaluation of limited irrigation strategies to improve water use efficiency and wheat yield in the North China Plain. *PLoS One*.

[44] Alghory A., Yazar A. (2019). Evaluation of crop water stress index and leaf water potential for deficit irrigation management of sprinkler-irrigated wheat. *Irrigation Science*.

[45] Ul-Allah S., Iqbal M., Maqsood S. (2018). Improving the performance of bread wheat genotypes by managing irrigation and nitrogen under semi-arid conditions. *Archives of Agronomy and Soil Science*.

[46] Sterling R., Neibling W. H. (1994). *Final Report of the Water Conservation Task Force*.

[47] Metin Sezen S., Yazar A. (2006). Wheat yield response to line-source sprinkler irrigation in the arid Southeast Anatolia region of Turkey. *Agricultural Water Management*.

[48] Hussain M., Farooq S., Jabran K., Ijaz M., Sattar A., Hassan W. (2016). Wheat sown with narrow spacing results in higher yield and water use efficiency under deficit supplemental irrigation at the vegetative and reproductive stage. *Agronomy*.

[49] Alghory A., Yazar A. (2018). Evaluation of net return and grain quality characteristics of wheat for various irrigation strategies under the Mediterranean climatic conditions. *Agricultural Water Management*.

[50] Meena R. P., Karnam V., Tripathi S. C., Jha A., Sharma G. P., Singh G. P. (2019). Irrigation management strategies in wheat for efficient water use in the regions of depleting water resources. *Agricultural Water Management*.

[51] Gao P., Duan T., Nan Z. (2018). The influence of irrigation frequency on the occurrence of rust disease (Melampsora apocyni) and determination of the optimum irrigation regime in organic Apocynum venetum_ production. *Agricultural Water Management*.

[52] Ahmadizadeh M., Nori A., Shahbazi H., Habibpour M. (2011). Effects of drought stress on some agronomic and morphological traits of durum wheat (*Triticum durum*) landraces under greenhouse condition. *African Journal of Biotechnology*.

[53] Huang Y., Chen L., Fu B., Huang Z., Gong J. (2005). The wheat yields and water-use efficiency in the Loess Plateau: straw mulch and irrigation effects. *Agricultural Water Management*.

[54] Zhang B., Li F., Huang G., Cheng Z., Zhang Y. (2006). Yield performance of spring wheat improved by regulated deficit irrigation in an arid area. *Agricultural Water Management*.

[55] Oury F. X., Godin C. (2007). Yield and grain protein concentration in bread wheat: how to use the negative relationship between the two characters to identify favourable genotypes?. *Euphytica*.

[56] Guttieri M. J., McLean R., Stark J. C., Souza E. (2005). Managing irrigation and nitrogen fertility of hard spring wheats for optimum bread and noodle quality. *Crop Science*.

[57] Dupont F. M., Hurkman W. J., Vensel W. H. (2006). Protein accumulation and composition in wheat grains: effects of mineral nutrients and high temperature. *European Journal of Agronomy*.

[58] Pompa M., Giuliani M. M., Giuzio L., Gagliardi A., di Fonzo N., Flagella Z. (2009). Effect of sulphur fertilization on grain quality and protein composition of durum wheat (Triticum durum Desf.). *Italian Journal of Agronomy*.

